# Faculty Training to Enhance Medical Education: Evaluating Laboratory Instructors at the National Autonomous University of Honduras (UNAH)

**DOI:** 10.7759/cureus.82535

**Published:** 2025-04-18

**Authors:** Génesis S Henriquez, Julia C Salinas Ulloa, Fernando J Caceres Carranza, Ginalizia Murillo Castro, Monica Fernanda Medina Guillen, German H Ramos, Kristopher J Varela Barrientos, Andrea J Rodas, David F Velásquez, Skarleth Bock, Javier S Asfura Caballero, Joyce Pineda, Jhiamluka Solano

**Affiliations:** 1 Medicine, Universidad Nacional Autónoma de Honduras, Tegucigalpa, HND; 2 Project Development, Honduran Medical Education Association (AEMH), Tegucigalpa, HND; 3 Departamento de Gestión Académica e Investigación, Hospital Escuela, Tegucigalpa, HND; 4 Obstetrics and Gynecology, Universidad Nacional Autónoma de Honduras, Tegucigalpa, HND; 5 Radiology, Secretaría de salud, Tegucigalpa, HND; 6 General Medicine, Hospital Lomas de Luz, La Ceiba, HND; 7 Project Development, Global Health Collaboration Project, Honduras, HND; 8 Resident Doctor Committee, Royal College of Physicians, London, GBR; 9 Education Committee, Academy of Medical Educators, Cardiff, GBR; 10 Cardiology, Scunthorpe General Hospital, Scunthorpe, GBR

**Keywords:** competency-based assessment, faculty development programs, medical edcuation, problem based learning (pbl), teaching methodologies

## Abstract

Background

Medical education is essential for training competent healthcare professionals capable of addressing evolving patient needs. Faculty development plays a crucial role in improving teaching quality and supporting competency-based education (CBE), particularly in Honduras, where reforms aim to modernize medical curricula. Despite a long history of medical training, formal, standardized faculty development programs remain limited, with many instructors relying on informal, unstandardized training. This study evaluates the impact of a structured faculty development program designed to strengthen teaching competencies and integrate CBE into medical education.

Objectives

This study aimed to identify gaps in faculty knowledge regarding self-directed learning (SDL), formative assessment (FA), reflective practice (RP), and problem-based learning (PBL); assess instructors’ interest in these methodologies; and evaluate competency improvements following a voluntary faculty development course.

Methods

A descriptive, cross-sectional study was conducted at two campuses of the National Autonomous University of Honduras (UNAH)-Ciudad Universitaria and Valle de Sula-with 219 active laboratory instructors. A voluntary learning needs assessment survey was completed by 141 instructors (64%) to evaluate baseline knowledge, perceived proficiency, and interest in SDL, FA, RP, and PBL. Based on the results, 107 instructors (49%) enrolled in a 10-week faculty development course and 45 instructors (20%) completed it. PBL was selected as the primary methodology due to its effectiveness in fostering critical thinking and alignment with UNAH’s educational reforms. The course included interactive sessions, competency-based assessments, and structured feedback. The primary outcome was the ability to design, facilitate, and assess a PBL session, evaluated using a standardized competency-based framework. Descriptive statistics were used to measure learning gains.

Results

The survey (N=141) revealed that 93% of instructors lacked formal pedagogical training, despite 83.8% having over two years of teaching experience. Institutional barriers - such as limited budget or development opportunities - contributed to this gap. Instructors rated their knowledge of SDL and FA as “good,” while RP was “average,” though interest in all methodologies was high. Post-training, participants demonstrated mastery of 91% of assessed competencies, with 70% struggling with PBL assessment tools. Inferential analysis using a Chi-square Test for Independence showed a significant association between training participation and competency achievement (χ² = 425.73, df = 75, p = 1.81 × 10⁻⁵⁰, Cramér’s V = 0.27). A majority (66.7%) rated the course a perfect 10/10, and 91.1% reported an improved understanding of educational methodologies.

Conclusions

This study underscores the urgent need for structured faculty development in Honduras. Implementing competency-based training earlier through policy changes, mandatory certification, and continuous professional development could enhance teaching quality, improve student engagement, and modernize medical education. Future research should include follow-up assessments to measure long-term retention, apply further inferential analysis, and explore challenges across specific competencies while addressing potential biases such as social desirability.

## Introduction

The 1998 World Conference on Higher Education emphasized the urgent need for continuous updates in competency-based learning to support the cultural, social, and economic development of society [1]. Medical education encompasses teaching methodologies, curriculum design, assessment strategies, and faculty development aimed at preparing healthcare professionals to meet patient needs holistically [2]. This field requires a high level of responsibility and adaptability, shifting the focus of learning toward competency-based approaches that integrate clinical skills with reflective practice (RP) and evidence-based decision-making [2]. Effective medical education depends on well-trained educators who not only master teaching strategies but also understand curriculum integration and competency evaluation [3]. This challenges the misconception that medical professionals automatically become competent educators, highlighting the essential need for formal training in medical education methodologies [3].

In Honduras, despite more than 50 years of offering a medical undergraduate degree at the UNAH, published research on medical education remains scarce. This scarcity may be attributed to limited institutional support, lack of funding, and the prioritization of clinical practice over pedagogical development. In 1995, a work session between semiology professors and pedagogy specialists from the Health Technology Unit (UTES) identified several deficiencies affecting teaching quality and student performance [4]. The key issues included the lack of integration between preclinical subjects in the curriculum, deficiencies in faculty training, low motivation among both educators and students and insufficient student preparation for advanced coursework [4]. However, despite recognizing these challenges, the 1995 curriculum reform implemented only minimal changes to faculty training, and no comprehensive, structured faculty development initiatives were introduced in subsequent years [5].

To address these long-standing challenges, outdated teaching methods must be replaced with evidence-based strategies that enhance knowledge integration and instructional effectiveness [6,7]. Improving the quality of medical educators is essential to fostering critical and reflective thinking in students, ultimately enhancing their academic and professional performance [6,7]. Achieving this requires comprehensive faculty development that extends beyond knowledge acquisition to include practical application, mentorship, and robust institutional support [4]. However, simply increasing educators’ knowledge is not sufficient to drive meaningful change; instead, it is crucial to implement strategies that promote a fundamental conceptual transformation in teaching methodologies from the earliest stages of an educator's career [8]. Learning needs assessments serve as a valuable tool for identifying gaps in faculty knowledge, allowing for the collection, organization, and interpretation of data to inform targeted interventions [9]. Ultimately, competency-based medical education cannot be effectively implemented without instructors who are fully equipped to develop, apply, and evaluate these competencies comprehensively, particularly in key areas such as self-directed learning (SDL), formative assessment (FA), RP, and problem-based learning (PBL) [1,10].

## Materials and methods

Study design and participants

We conducted a descriptive cross-sectional study with elements of an educational intervention and quasi-experimental design to assess the learning needs of laboratory instructors and implement a comprehensive faculty training program focused on innovative medical education methodologies. The quasi-experimental component involved pre- and post-assessments to evaluate competency improvements following training. The laboratories at the National Autonomous University of Honduras (UNAH) Ciudad Universitaria (CU) and Valle de Sula (VS) campuses primarily serve preclinical students and support various subjects, including Embryology, Histology, Physiology, Pathophysiology, and Pharmacology, by integrating medical students under faculty supervision. Laboratory instructors differ from regular faculty members in that they primarily facilitate practical learning and hands-on experiences more frequently than traditional lectures.

Selected participants were active laboratory instructors from UNAH in either of both campuses and subjects that use laboratories. All eligible instructors were invited to participate, and no sampling method was used. To deliver the training course, we trained 10 facilitators with former experience as laboratory instructors and demonstrated teaching proficiency through prior formal pedagogical training [11]. Facilitators were selected based on their teaching experience and competency in PBL facilitation. UNAH Faculty of Medical Sciences (FCM) Biomedical Research Ethics Committee (CEIB) reviewed and approved the study protocol (IRB Registration No. 00003070. APPROVED 036-2023), adhering to the Helsinki Declaration.

Learning needs assessment

The survey comprised nine sections, including socio-demographic information, the laboratory where instructors taught, semesters/years of teaching experience, and prior training in teaching. Additionally, a modified Self-Directed Learning Readiness Scale (SDLRS) assessed the need for training based on prior knowledge and interest in learning about medical education methodologies such as RP, SDL, FA, EF, and PBL. The SDLRS was adapted from a validated instrument to better align with medical education contexts. Responses were self-reported using a 5-point Likert scale ranging from “Strongly Disagree” to “Strongly Agree.”

Training

The training course lasted 10 weeks, comprising interactive teaching sessions that covered key aspects of medical education. Sessions explored both traditional and student-centered methodologies, with a strong emphasis on RP, SDL, FA, EF, and PBL. RP training involved documenting, applying, and evaluating learning through reflective models. SDL focused on conceptual frameworks, practical applications, and methods for guiding students as self-directed learners. FA included core principles, identifying learning needs, practical applications, and evaluation techniques. EF training covered its theoretical foundation, types, and practical applications. PBL, chosen as the primary teaching methodology due to its effectiveness in fostering critical thinking, was integrated through case studies and role-playing, employing small-group learning with groups of 8-10 participants per session.

To monitor effectiveness, participants were assessed through continuous formative evaluations during each module, including observation of teaching practice and competency demonstrations. Each participant led a PBL session as a capstone activity, with their facilitation skills evaluated by trained observers.

Assessment

The training concluded with an assessment and personalized feedback session. The primary assessment protocol included self-assessment and trainer evaluation, using a standardized competency-based rubric developed and validated through expert consensus. Each competency was rated on a 5-point Likert scale ranging from “Highly Disagree” to “Highly Agree.” To pass the course, the instructor needed to achieve “Agree” or “Highly Agree” ratings in at least 31 (75%) of the 42 evaluated competencies, a threshold based on established standards at UNAH for passing courses/classes. Trainers provided constructive feedback for competencies not met.

Feedback form

Participants completed an anonymous feedback form after their one-on-one feedback session with the trainer. The form included both open-ended questions and Likert-scale responses to gather qualitative and quantitative insights on the training experience. The feedback aimed to evaluate participants’ perceptions of knowledge improvement and course effectiveness, as well as to identify areas for further program enhancement. All responses were self-reported, and no external validation was performed.

Data collection and statistics

Data from the initial survey, final assessments, and course feedback forms were collected using Google Forms and analyzed using SPSS. Descriptive statistics summarized participant characteristics and survey responses, while inferential statistics, including chi-square tests for competency comparisons and paired t-tests for pre- and post-training assessments, were conducted. The reliability of the survey responses was tested using Cronbach’s alpha (α = 0.85). Data handling procedures ensured anonymity and data integrity, adhering to institutional data management policies.

## Results

Participants

**Figure 1:**
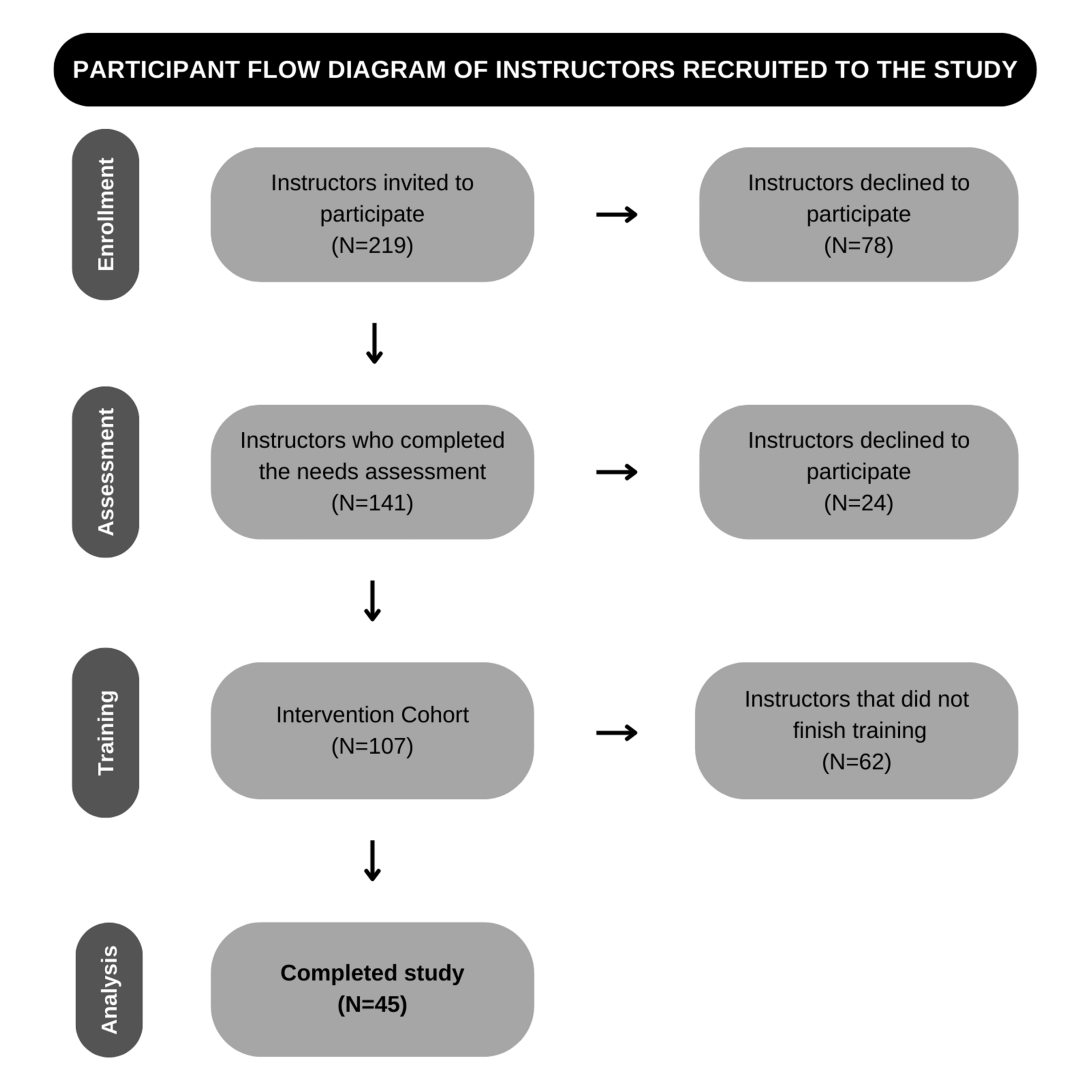
Participant flow diagram of instructors recruited to the study.

All 219 active instructors were invited to participate. The participant flow diagram is shown in Figure 1. A total of (n=141) participants completed the initial needs assessment survey. Of those, 107 (75%) agreed to enroll in the training intervention, but only 45 (32%) completed the full course. The dropout rate was substantial, with 62 (58%) instructors not finishing the training. Potential reasons for this attrition could include scheduling conflicts, high workload demands, or disengagement. Despite this, the retention rate among those who enrolled in the training remained meaningful, with 45 completing the study. This suggests that while initial participation faced challenges, those who remained engaged, probably found the program valuable.

Learning needs assessment

Demographics

The cohort was evenly distributed by gender, with 70 (49.7%) female and 71 (50.3%) male instructors. In terms of institutional affiliation, 88 (62.4%) instructors were from the UNAH - CU Campus, while 53 (37.6%) were from the UNAH - VS Campus. Participants were also distributed across different academic years, as these instructors are also medical students who serve as educators for students in lower years. Specifically, 37 (26.2%) were in their third year, 43 (30.5%) in their fourth year, 34 (24.1%) in their fifth year, and 27 (19.2%) in their sixth year.

Regarding the subjects taught, 34 instructors were involved in teaching Embryology, 59 instructors teaching Histology, 39 instructors teaching Physiology, and 22 instructors teaching Pathophysiology and Pharmacology. While some instructors specialized in a single subject, others taught across multiple disciplines.

Experience levels varied significantly, with 118 (83.8%) instructors having more than two years of experience. However, only 10 (7%) had received any form of teaching training, which included formal or informal certifications, faculty development workshops, and prior pedagogical training sessions. This may be due to lack of funding, or lack of trained tutors.

Themes

Most respondents rate their knowledge (Table 1) as “Average” or “Good,” with low “Excellent” ratings, indicating limited expertise or training. RP has the highest “Poor” perception 41 (29%). Despite this, (Table 2) over 110 (78%) are “Highly Interested” in all topics, especially medical education 124 (90%) and SDL 117 (84%), highlighting a gap between perceived knowledge and the demand for further education.

**Table 1:** Results of the needs assessment (n=141).

	Perception of Knowledge							
	Poor		Average		Good		Excellent	
	N	%	N	%	N	%	N	%
Medical Education	5	(3)	54	(38)	76	(55)	6	(4)
Reflective Practice	41	(29)	68	(48)	30	(22)	2	(1)
Self-Directed Learning	9	(6)	62	(44)	65	(47)	5	(3)
Formative Assessment	17	(12)	52	(38)	62	(44)	9	(6)
Effective Feedback	20	(14)	58	(42)	54	(38)	9	(6)
Problem-Based Learning	4	(2)	53	(37)	77	(56)	7	(5)

**Table 2:** Results of the needs assessment (n=141).

	Interest in Learning About It							
	Not interested		Somewhat interested		Interested		Highly interested	
	N	%	N	%	N	%	N	%
Medical Education	5	(3)	4	(2)	8	(5)	124	(90)
Reflective Practice	4	(2)	9	(6)	18	(14)	110	(78)
Self-Directed Learning	2	(1)	9	(6)	13	(9)	117	(84)
Formative Assessment	5	(3)	7	(5)	16	(11)	113	(81)
Effective Feedback	4	(2)	6	(4)	21	(16)	110	(78)
Problem-Based Learning	5	(3)	3	(2)	17	(12)	118	(83)

Tables 1-2 present both knowledge perception and interest in learning, and a clear trend emerges: instructors who rated their knowledge as “Poor” often showed higher interest in learning. For example, despite the low perceived knowledge in RP, 110 (78%) expressed a high interest in improving their skills. Similarly, SDL saw 117 (84%) highly interested despite moderate knowledge ratings, highlighting a gap between self-perceived competency and the demand for further training.

Most respondents rated their knowledge as “Average” or “Good,” with limited “Excellent” ratings, indicating moderate expertise. RP had the highest “Poor” perception at 41 (29%). The Readiness Scale used in the study does not simply contrast high or low knowledge and interest but instead generates a quotient that determines the need for a tutor. Given that most participants rated their knowledge as average to good and their interest as high, this perspective helped inform a purposeful training program tailored to their needs and willingness to engage in further education.

End-of-training assessment

The end-of-course assessment framework showed that most instructors achieved over 38 (91%) of the 42 competencies assessed (Table 3). However, it is important to clarify that this means 91% of the total competencies were successfully achieved across participants, rather than 91% of instructors passing. Given the requirement to achieve “Agree” or “Highly Agree” in at least 31 (75%) competencies to pass, all students who finished the course met the threshold.

**Table 3:** End-of-training competency assessment framework (n=45).

Competency Area	Highly Disagree		Disagree		Agree		Highly Agree	
	N	%	N	%	N	%	N	%
Reflective Practice								
Able to reflect	0	(0)	0	(0)	15	(33)	30	(67)
Able to identify areas for improvement	0	(0)	0	(0)	23	(52)	22	(48)
Identifies the benefits of reflective practice	0	(0)	0	(0)	9	(20)	36	(80)
Self-Directed Learning								
Follows the steps of self-directed learning (SDL)	0	(0)	0	(0)	19	(42)	26	(58)
Facilitates the learning process	0	(0)	9	(20)	26	(58)	10	(22)
Formative Assessment								
Defines learning objectives	0	(0)	0	(0)	15	(33)	30	(67)
Provides feedback at each step	0	(0)	9	(20)	26	(58)	10	(22)
Creates strategies to improve learning	0	(0)	4	(9)	26	(58)	15	(33)
Effective Feedback								
Feedback is specific	0	(0)	13	(30)	10	(22)	22	(48)
Feedback is provided on time	0	(0)	4	(9)	28	(62)	13	(29)
Feedback is constructive	0	(0)	4	(9)	28	(62)	13	(29)
Feedback is clear	0	(0)	0	(0)	22	(48)	23	(52)
Problem-Based Learning (PBL): Case selection								
Important	0	(0)	0	(0)	10	(22)	35	(78)
Realistic	0	(0)	0	(0)	9	(20)	36	(80)
Engaging	0	(0)	0	(0)	17	(38)	28	(62)
Challenging	0	(0)	2	(4)	2	(4)	41	(92)
Instructional	0	(0)	0	(0)	10	(22)	35	(78)
Case Organization								
Learning points	2	(4)	10	(22)	0	(0)	33	(74)
Differential diagnosis	0	(0)	0	(0)	2	(4)	43	(96)
Relevant clinical findings	0	(0)	0	(0)	12	(26)	33	(74)
Clinical reasoning	0	(0)	2	(4)	15	(33)	28	(63)
Opportune text and image aid	0	(0)	2	(4)	17	(38)	26	(58)
Assigning roles	0	(0)	5	(11)	10	(22)	30	(67)
Presenting clinical, laboratory, and imaging investigations findings	0	(0)	0	(0)	15	(33)	30	(67)
Assessment Framework								
Defines evaluation criteria and levels of mastery	10	(22)	26	(58)	2	(5)	2	(5)
Develops rubrics	10	(22)	26	(58)	7	(15)	7	(15)
Overall	0.57	(0.01)	5	(9)	15	(33)	26	(58)

Strengths were particularly evident in PBL case selection and organization, where instructors demonstrated strong competency. However, challenges emerged in EF, where disagreement rates reached 13 (30%), and in the assessment framework, where 10 (22%) disagreed and 26 (58%) highly disagreed with their ability to define evaluation criteria and develop rubrics. This suggests that instructors struggled with rubric design and competency-based assessment, potentially due to a lack of prior exposure to structured evaluation methods. Additional targeted training or practice opportunities in these areas could improve performance.

**Figure 2:**
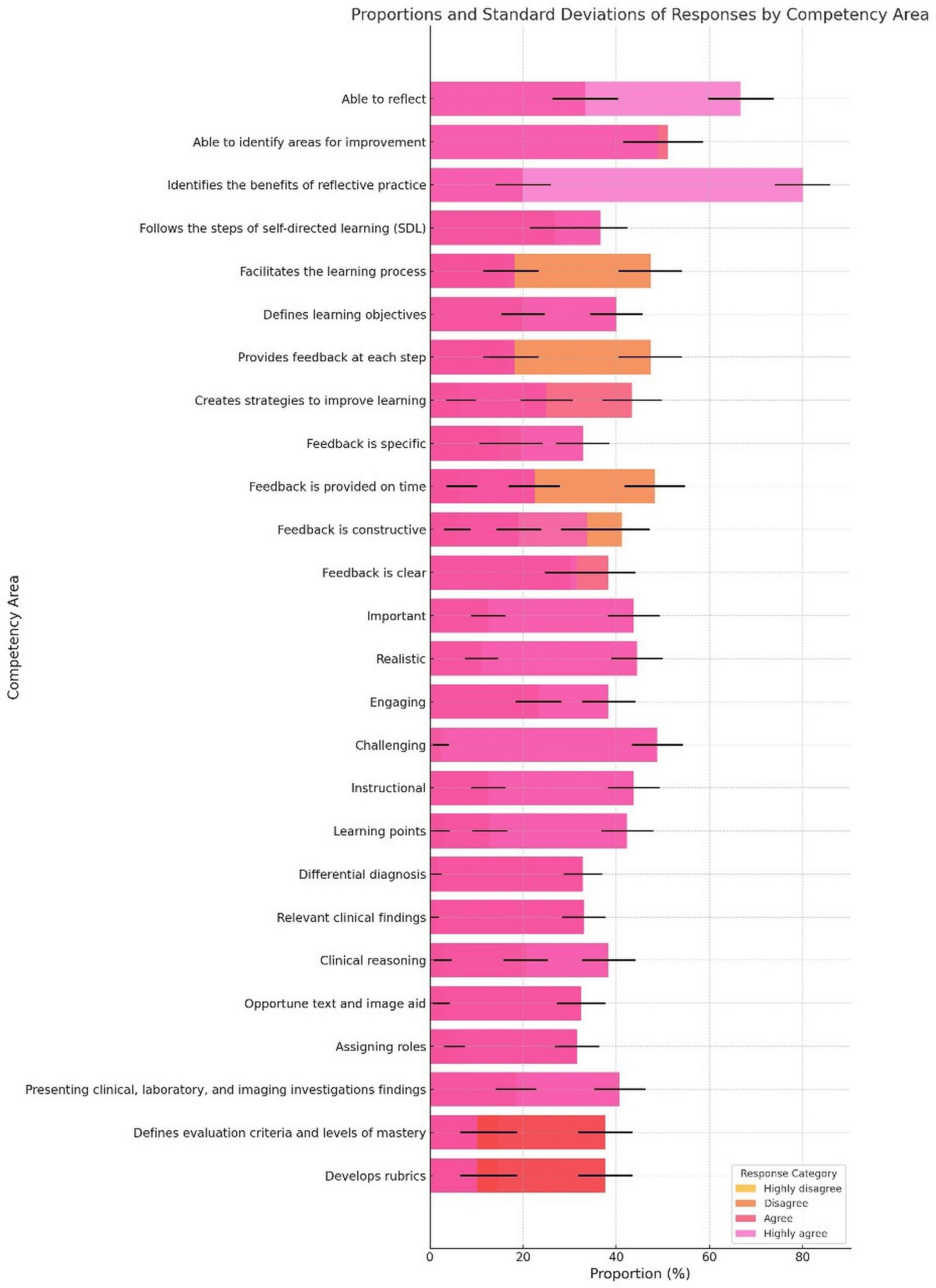
Standard deviation between each competency assessed. Each bar represents the percentage of responses per category, while the black horizontal lines over the bars indicate the standard deviation.

Figure 2 illustrates the proportions of participants who responded across four levels of agreement - Highly disagree, Disagree, Agree, and Highly agree - for each assessed teaching competency area following the faculty development program. Each bar represents the percentage of responses per category, while the black horizontal lines over the bars indicate the standard deviation, highlighting variability in responses within each competency. To evaluate the relationship between participation in the faculty development program and competency achievement across various teaching domains, a Chi-square Test for Independence was performed. The analysis yielded a chi-square statistic of 425.73 with 75 degrees of freedom, and a p-value of 1.81 × 10⁻⁵⁰, indicating a statistically significant association (p < 0.05). The Cramér’s V value of 0.27 suggests a moderate effect size, highlighting a meaningful relationship between the training intervention and improved competency outcomes. This supports the fact that faculty involvement in structured development programs is significantly associated with improved teaching performance across multiple competency areas.

Course feedback

The majority of instructors rated the course highly in terms of its effectiveness in improving their knowledge of medical education methodologies, with 30 (66.7%) giving it a perfect score of 10 out of 10. In comparison, 11 (24.4%) rated it 9 out of 10. Following the training, 41 (91.1%) of participants reported a clear understanding of all concepts and methodologies taught. However, a small percentage of instructors (1-3 individuals per category) reported difficulties in specific areas, such as RP, FA, and PBL assessment tools. Some of these difficulties were addressed in real-time during the course and the door remained open for further aid.

Additionally, 40 (88.9%) of instructors acknowledged that trainers effectively facilitated learning rather than merely delivering information. The most effective aspects of facilitation included interactive discussions, real-world case applications, and structured peer collaboration, which helped reinforce key concepts.

Regarding course resources, most participants found interactive sessions, assessments, and feedback essential. Participants found the facilitation highly effective, praising the clear, interactive, and practical approach that enhanced their understanding of teaching methodologies in medical education. They appreciated the structured format, combining theory with practice, and valued the instructor’s ability to simplify complex topics while encouraging participation. Many felt the course exceeded their expectations, providing tools to improve their teaching and laboratory sessions. While some wished for more depth in certain topics, the overall consensus was that the course was enriching, shifted their perspective on education, and equipped them with practical strategies they are eager to apply.

However, a significant portion of instructors regarded assignments as unnecessary. This raises an important question: were assignments perceived as redundant, too difficult, or not applicable to their teaching practice? Even though assignments were deemed voluntary for their own entertainment and aid to understand the subject prior to our open discussion sessions; addressing this concern could involve redesigning assignments to align more closely with instructors' real-world teaching contexts rather than reducing them outright.

## Discussion

Traditional teaching approaches often distance students from active learning, reducing them to passive spectators with minimal responsibility for their own education [8]. The findings of this study align with existing research on faculty training in medical education, reinforcing the critical need for structured educator development programs. Similar studies in Mexico have reported significant deficiencies in faculty training, particularly in assessment methodologies and competency-based education [6]. This mirrors the high percentage of untrained instructors (131 or 93%) observed in this study. Additionally, international research highlights that faculty development programs significantly improve teaching effectiveness, with studies from Canada, Australia, and the United States demonstrating that structured training enhances SDL, FA, and PBL implementation [1]. The high competency scores (38 or 91%) achieved by participants in this study reflect similar trends found in research on faculty development initiatives, where instructors trained in student-centered methodologies show measurable improvements in their teaching strategies and engagement levels [1,12].

However, challenges in RP are noted as an area of average understanding among participants. This aligns with previous studies indicating that reflection is one of the more difficult competencies to develop [6,8,13]. Research suggests that effective reflection requires ongoing practice and mentorship rather than short-term training, which could explain why some participants in this study still struggled in this area despite overall improvements [14,15]. To improve RP adoption, institutions could implement structured reflection templates and peer feedback mechanisms, providing participants with clear guidance on how to approach and improve their RP [14,15]. Mentorship programs could also play a crucial role in this context, providing sustained support for participants as they practice reflection in real-life teaching environments [14,15].

Additionally, the finding that interactive sessions and structured feedback were the most valuable resources, while assignments were perceived as less necessary, aligns with studies emphasizing the importance of active learning strategies over passive assessment tasks in faculty training [1,12]. To further strengthen the impact of faculty training, incorporating more mentorship and extended workshops that focus specifically on interactive methods and feedback mechanisms could address areas where participants feel they need further support [1,12].

Overall, these results reinforce global trends advocating for competency-based faculty development, highlighting both the successes and challenges of implementing medical education training programs. Future research should explore the long-term retention of these competencies and their impact on student learning outcomes, as seen in studies where faculty training led to improved student performance and engagement [6,12,16].

To date, there’s evidence that faculty efforts have successfully extended training to approximately 51 additional laboratory instructors through a 50-hour pedagogy course [17].

Transforming medical education

Faculty must undergo comprehensive training to integrate evidence-based teaching methodologies into medical education effectively [4,6]. Research indicates that 40 to 80 hours of structured training are necessary to create meaningful changes in instructional practices [4,6]. Structured assessments, such as self-perception questionnaires, are commonly used to identify learning gaps [4]. When a significant knowledge gap is detected, facilitators play a crucial role in guiding educators toward improved teaching strategies [4].

There is a lack of published research on medical education and faculty training in Honduras. No documented studies assess the learning needs of medical instructors in the region. However, research in Mexico highlights similar challenges, particularly in faculty training and assessment methodologies. The lack of structured faculty development has resulted in low-quality, outdated teaching methods that persist in medical education [4]. The absence of comprehensive faculty development programs is a notable gap in both Honduras and similar Latin American contexts. While there are broad institutional barriers such as limited funding and resistance to changing long-established methods, adapting faculty development strategies from countries like Mexico, where training programs have been implemented with some success, could provide valuable insight for Honduras moving forward.

The first step in improving medical education is adopting teaching methodologies with proven effectiveness. Countries such as England, Canada, Australia, and the United States have successfully transitioned to competency-based medical education (CBME), which focuses on skills development, feedback integration, and active learning [1,18]. Research has shown that curricula incorporating structured feedback mechanisms lead to substantial learning gains [8]. Additionally, students exposed to PBL develop greater cognitive processing skills than those in conventional lecture-based programs [18]. These students also adopt SDL and long-term self-monitoring habits, and they demonstrate improved performance [16,18].

Traditional teaching methods

Medical education is unique in that it extends beyond standard higher education due to its technical foundation in biosciences, requiring specialized teaching approaches. Despite this, many institutions have relied on traditional teaching models that assume physicians inherently possess teaching skills. As a result, faculty members, despite spending a significant portion of their careers teaching, often lack formal training in pedagogical strategies, assessment techniques, and student-centered learning approaches.

Outdated methodologies, such as summative assessments, lecture-based learning, and passive student participation, continue to dominate medical education. This leads, among other things, to a lack of integration in teaching, neglecting the development of critical thinking. Although some institutions attempt to modernize teaching through short refresher courses, achieving lasting change remains a challenge. Transforming the mindset of an instructor who has relied on repetitive, traditional methods for years requires more than a brief training session - it necessitates a fundamental shift in teaching philosophy [4,6].

Student-centered learning

Student-centered methodologies, in contrast, focus on critical thinking with learning, teaching, and assessment processes that are constantly improving their quality [8]. These methods utilize structured assessments, performance-based rubrics, and real-world applications to ensure that students build competencies in a meaningful, hands-on manner [8]. This commits the students to building their own competencies through real-life situations, and with that, their learning revolves around significant experiences led by the teacher [8,12].

Student-centered learning approaches enhance competency by engaging students in real-life scenarios, with instructors acting as facilitators. Methods like RP, SDL, EF, FA, and PBL bridge theory and practice. These strategies are most effective when aligned with students’ individual learning styles, which influence study habits and academic performance-especially in digital and post-pandemic settings. As noted, learning styles impact study duration and success [19].

Limitations

This study has several limitations that should be considered when interpreting the findings. First, the sample size was relatively small (n=45) for the full training cohort, which may limit the generalizability of the results to all medical instructors at UNAH or other institutions in Honduras. Additionally, the study primarily relied on self-reported data from surveys and feedback forms, which are subject to response bias-instructors may have overestimated their understanding or engagement with the course. There was also a lack of control group for comparison.

Another limitation is the short duration of the training intervention. While participants demonstrated immediate improvements in their competency scores, the study did not include a long-term follow-up to assess whether these skills were retained or applied effectively in real teaching settings. Furthermore, while the study focused on medical education methodologies, it did not assess how these newly acquired skills translated into student learning outcomes, which is a key measure of educational effectiveness. This study acknowledges the absence of comparative analysis and potential biases thereafter.

Lastly, the study was conducted at a single institution (UNAH) across two campuses, which may not fully capture the diversity of challenges faced by medical educators in other regions or institutions.

## Conclusions

This study highlights the urgent need for faculty training in medical education, as most instructors lacked formal training despite several semesters of teaching experience. Many participants rated their knowledge of methodologies as “average/good,” yet their lack of training didn’t reflect a lack of interest, as all methodologies received high scores for further learning. The training program effectively enhanced teaching competencies, with participants mastering the greater part of the evaluated competencies. The course was well-received, with a predominant perfect rating and a strong understanding of the concepts. Interactive sessions, structured feedback, and reading materials were valued, while assignments were seen as less essential. Addressing faculty learning gaps early in instructors' careers is crucial for improving teaching quality and student outcomes.

This study contributes to the literature by providing valuable data on faculty training needs and challenges in Honduras, a country with limited research in this area. It also adds to the growing body of work advocating for structured faculty development programs, particularly in regions where such initiatives have been underexplored. The findings echo global trends emphasizing the importance of competency-based faculty development, while also highlighting the specific challenges faced in a developing educational context like Honduras. Future research should expand the sample, incorporate objective assessments, and conduct longitudinal studies to evaluate the long-term impact of faculty development programs.

## Appendices

The assessment rubric is displayed in Table 4.

**Table 4:** Assessment rubric.

	Highly disagree	Disagree	Agree	Highly agree
Reflective Practice				
Chosen model: Borton				
The facilitator, after the session, is able to reflect, according to the chosen model, on his or her actions and decisions during the session.				
The facilitator is able to identify for himself areas in which he could improve.				
The facilitator understands the benefits of using reflective practice for this session and future practices.				
Self-Directed Learning				
To develop his notes, the facilitator followed the steps of self-directed learning:				
What do I want to know?				
How much do I already know about that?				
How do I learn best?				
What resources can I use?				
How, where, and when will I study?				
Opening				
What did I learn?				
What should I review?				
The facilitator was able to support and guide the learning process rather than simply handing out information.				
Formative evaluation				
The facilitator is able to define the learning objectives of a session.				
The facilitator is able to provide feedback to the participants at each step.				
The facilitator is able to analyze a strategy that corrects the deficiencies found during this session.				
Effective feedback				
The facilitator is able to provide effective feedback.				
Your feedback is specific.				
Your feedback is given on time.				
Your feedback is constructive (provides ways to solve)				
Your feedback is clear.				
Problem-Based Learning				
The facilitator is able to search for a suitable case for the session.				
Important				
Content appropriate for the intended audience				
Content that matches learning objectives				
An explicit narrative setting for the case				
Realist				
Authentic situations and characters				
Sufficient irrelevant information to reflect clinical realities				
Gradual and well-paced dissemination of content				
Attractive				
Rich content that contributes to the complexity of the case				
Branch that allows students to determine the progress or outcome in the case				
Challenging				
Appropriate level of difficulty or structure for the intended audience				
Appropriate disease prevalence or presentation for the intended audience				
Related to other cases found by the target audience				
Instructive				
The case activates the participants' prior knowledge.				
The case integrates material found using other methodologies.				
The case stimulates the development of learning problems by students.				
The facilitator is able to organize the information of the case found				
Potential learning problems				
List of possible differential diagnoses				
Positive and negative findings relevant to the case according to differential diagnoses.				
Questions or guidelines that encourage clinical reasoning				
Timely textual and image reminders				
The facilitator is able to delegate the roles of the participants in the session.				
The facilitator is able to present and present the clinical, laboratory, and imaging findings to the participants in order to reach the expected diagnosis				
Evaluation Rubrics				
The facilitator is able to define the evaluation criteria.				
The facilitator is capable of developing an evaluation rubric consistent with his or her evaluation criteria and level of mastery.				
